# A Narrative Review of Mesh Suture in Abdominal Wall Reconstruction: Biomechanics, Early Outcomes, and Proposed Clinical Algorithm

**DOI:** 10.3389/jaws.2025.15452

**Published:** 2025-11-27

**Authors:** Megan M. Perez, Gregory A. Dumanian, Paige N. Hackenberger, David Ross, Jonah Stulberg, Michael Shapiro

**Affiliations:** 1 Division of Plastic Surgery, Northwestern Feinberg School of Medicine, Chicago, IL, United States; 2 Emeritus Plastic Surgeon, St. Thomas’ Hospital, London, United Kingdom; 3 University of Texas, Houston, TX, United States; 4 Department of Surgery, Northwestern Feinberg School of Medicine, Chicago, IL, United States

**Keywords:** abdominal wall closure, contaminated wounds, mesh, mesh complications, hernia

## Abstract

**Background:**

Suture repair of abdominal wall defects is prone to failure due to suture pull-through. In contrast, planar mesh reinforcement improves durability but is limited by increased foreign body burden, need for additional tissue dissection, and challenges in contaminated fields. Mesh suture offers a potential alternative combining both the ease of suture repair with improved load distribution and early tissue integration, characteristics of planar mesh repairs. This review summarizes the biomechanical rationale, histologic characteristics, and early clinical experiences with mesh suture to date.

**Methods:**

A narrative review of preclinical and clinical literature regarding mesh suture was performed using a targeted search of PubMed and Google Scholar with key terms (“mesh suture” or “Duramesh”). Studies were included if they evaluated mesh suture in biomechanical, preclinical or clinical contexts. A proposed clinical algorithm based on institutional experiences is presented to illustrate patient selection and technique.

**Results:**

Preclinical studies demonstrate favorable mechanical performance and early fibrovascular incorporation. Early clinical data from registries and institutional cohorts suggests mesh suture is feasible even in contaminated settings with outcomes that compare to both standard suture and planar mesh repairs.

**Conclusion:**

Mesh suture may offer a reinforcement strategy that balances mechanical support with tissue preservation in abdominal wall reconstruction. Current clinical evidence remains preliminary, and additional prospective, randomized studies are needed to more definitely evaluate its clinical performance over time.

## Introduction

Incisional hernia is a common complication of abdominal wall surgery, affecting up to 20% of closures and more than half of high-risk patients [1–3]. A key cause of repair failure is suture pull-through, where high pressure at the suture-tissue interface (STI) causes fascia to tear, leading to dehiscence or hernia [4–8]. Historically, repair durability has been improved through mesh reinforcement that distributes forces over a larger surface area increasing tensile strength [4, 9–12]. However, planar mesh introduces risks of extrusion, fistula, seroma, hematoma, pain, and delayed healing. Wide tissue dissection increases risk of wound complications further limiting its prophylactic use of mesh for laparotomy closure. These challenges prompted interest in alternatives that balance support with tissue preservation.

Mesh suture (Duramesh, Mesh Suture Inc., Chicago, IL) has emerged as a middle ground between suture-only closure and planar mesh. Mesh suture is made from braided and bonded polypropylene filaments that distribute mechanical load, thus reducing focal pressure at the STI and the likelihood of suture pull-through [5, 13, 14]. Biomechanical studies show greater tensile strength and less failure compared to monofilament suture [5]. Furthermore, its macroporous structure promotes early fibrovascular ingrowth, potentially enhancing strength and resistance to infection [15]. Additional reconstructive uses exist, making mesh suture a versatile adjunct in clinical surgery[8, 16–18].

To identify relevant literature for this narrative review, a targeted search of PubMed and Google Scholar was performed for the period 2010 through July of 2025 using the terms (“mesh suture” OR “Duramesh”). Articles were screened for full text and included if they specifically evaluated mesh suture (Duramesh) in biomechanical, preclinical or clinical studies. Although basic inclusion/exclusion criteria were applied as mentioned above, no formal systematic inclusion or exclusion framework was used given this was not a systematic review. This narrative review provides a comprehensive summary of the current literature on mesh suture, including its biomechanical properties, histologic response, and early clinical applications across a range of contexts. A proposed clinical algorithm is presented based on institutional experiences. Although current findings should be interpreted with caution given the early stage of clinical evidence, mesh suture appears to be a promising alternative in select cases.

## Biomechanical Foundations

At the STI, local tissue pressure is the axial force along the suture divided by the contact area. When this pressure exceeds the strength of fascia or tissue, suture pull-through occurs. Theoretic and modeling studies show fine-caliber sutures create high focal pressure, whereas larger or flatter sutures distribute load more evenly [19]. Thus, repair durability depends not only on suture strength but also on geometry, distribution of contact points, tissue quality, and biologic integration [19]. The “small-bites” technique leverages this principle [20]. Mesh suture was designed to optimize these factors. Constructed from either 12 or 18 bonded polypropylene filaments, mesh suture increases STI contact area about eight-fold as compared to a monofilament [15]. This structure enhances load-sharing, distributes mechanical stress, and provides a scaffold for biologic repair. Like barbed suture that functions as multiple linear anchors by resisting displacement along the axis of pull, mesh suture maximizes tissue engagement through friction and integration rather than tension alone [21]. Unlike barbed sutures whose rigid unidirectional projections can lead to focal stress concentrations, mesh suture offers a flexible, porous structure that balances mechanical reinforcement with biologic incorporation.

Preclinical models demonstrate greater pull-through resistance, tensile strength, and stress distribution with mesh suture versus monofilament [5, 22, 23]. In a simulated burst model, mesh suture resisted cyclic loading failure better than monofilament [24]. In a porcine model, it maintained suture tension and improved force redistribution with the small-bite technique [25]. Collectively, these findings support mesh suture’s mechanical integrity under physiologic and supraphysiologic stress. A distinguishing feature of mesh suture is its deformation under load: filaments flatten into a ribbon-like shape, increasing STI contact area and reducing peak pressure, as confirmed by finite element modeling [15, 26].

Mesh suture also demonstrates favorable histology. In a porcine model, near-complete fibrovascular ingrowth with collagen, fibroblasts, and capillaries surrounded the filaments by day eight [14]. In contrast, monofilament closures showed poor engagement and small herniations. High porosity and surface area of foreign bodies are associated with more favorable responses, promoting scarring sufficient for durability without bridging fibrosis [27].

## Mesh Strips: A Historical Precursor

Before mesh suture became clinically available, Lanier et al. described the use of “mesh strips” for abdominal wall closure as a natural outgrowth from pre-clinical studies [7]. The technique involved cutting two centimeter wide strips from large-pore, soft polypropylene mesh and passing them in a running or interrupted fashion using a number 1 polypropylene suture/needle tied to the end of the strip as an introducing agent. Mesh strips aimed to combine the strength and surface area of planar mesh with the technical flexibility and simplicity of suture-based closure, particularly in contaminated or complex fields where traditional planar mesh might pose unacceptable risks.

Lanier et al. first described a series of complex abdominal wall reconstructions using mesh strips in place of monofilament suture for fascial closure, with a hernia recurrence rate of 4.1% [7]. Building on this experience, Dumanian et al. evaluated outcomes in a cohort of contaminated incisional midline hernia repairs in 48 patients closed with mesh strips [28]. In this single-center study, the one-year hernia recurrence rate was 6%, lower than expected in such contaminated settings based on national benchmarks [28]. A mesh strip study for umbilical hernia repairs revealed only 1 of 33 patients to have a recurrence at 3 years after implantation [29]. A recent systematic review by Nip et al. summarized outcomes of both mesh suture and mesh strips, demonstrating that these constructs reduced the incidence of incisional hernia after abdominal wall closure or ventral hernia repair [30]. These studies highlighted the conceptual promise of wider, load-sharing suture constructs but also revealed the practical limitations of mesh strips, which lacked standardization in preparation and application. Specifically, the brand of mesh used for strip creation, strip width, and the method of passing strips through tissues with the broad aspect remaining orthogonal to the direction of force to maximize their potential in limiting suture pull-through may affect reproducibility and performance. Despite these challenges, global clinical experience with mesh strips provided compelling proof-of-concept for the approach. The subsequent development of mesh suture directly addressed these shortcomings, offering a pre-manufactured, standardized construct with consistent diameter, porosity, knot profile, and handling characteristics optimized for surgical use.

## Clinical Applications and Early Outcomes

Mesh suture has been adopted across a broad range of surgical contexts, including abdominal wall laparotomy closure, hernia repair, and aesthetic abdominal wall contouring (Table 1).

**TABLE 1:** Early Clinical Studies using Mesh Suture.

Citation	Study Type	N	Indications	Key Findings	Mean Follow-up
Hackenberger et al. [8]	Multicenter retrospective registry	314	Abdominal wall closure	SSI 6.1% SSE 11.8% Recurrence 0.6%	81.9 days
Hackenberger et al. [16]	Case series	25	DIEP flap donor site closure	SSI 4% SSE 16% Hernia 0%	Not stated
Marangi et al. [17]	Prospective cohort	33	Rectus diastasis repair, abdominoplasty	SSI 1.5% Wounds 6.2% Recurrence 0%	182 days
Perez et al. [31]	Multicenter retrospective registry	862	Abdominal wall closure	SSI 9.0% SSE 11.8% Recurrence 4.8%	193.5 days
Perez et al. [32]	Multicenter retrospective registry	47	Rectus diastasis repair with and without hernia, abdominoplasty	SSI 2.1% Wounds 8.5% Recurrence 2.1%	354 days
Perez et al. [33]	Multicenter retrospective registry	51	Contaminated midline hernia repairs	SSI 15.7% SSO 23.5% Recurrence 8.2%	389 days
Quattrone et al. [18]	Single-center retrospective series	63	Ventral hernia repair	SSI 6.3% SSE 15.9% Recurrence 4.8%	45 days
Takaya et al. [34]	Case report	1	Pediatric abdominal wall reconstruction	Tension-free closure, no complications or recurrence	90 days

The largest study of mesh suture outcomes to date included 1,111 patients treated by 86 surgeons across 11 specialties [31]. Among 862 full-thickness abdominal wall closures performed without concomitant use of planar mesh, the 90-day SSI rate was 9.0%, fascial dehiscence 1.0%, and hernia recurrence 4.8% at a median follow-up of 193 days. Mesh suture was used across all wound classes (CDC I-IV) with no association between contamination and recurrence. Chronic draining sinus (0.3%) and enterocutaneous fistula (0.2%) rates were low, consistent with earily registry data [31]. [8]. In a cohort of 63 ventral hernia repairs, Quattrone et al. reported a 4.8% recurrence and 15.9% 90-day SSE rate, supporting mesh suture as a technically feasible option in high-risk or contaminated fields [18]. Marangi et al. demonstrated favorable short-term outcomes in rectus diastasis >6 cm, and in 25 DIEP flap reconstructions, SSI was 4.0% with no chronic complications [16, 17]. A recent pediatric case report also achieved closure of a massive abdominal wall defect without planar mesh [34]. Together, these findings indicate that mesh suture achieves infection and wound complication rates comparable to historical benchmarks while maintaining low rates of chronic mesh-related morbidity in the short term.

## Positioning Among Comparative Techniques

The clinical role of mesh suture remains evolving and must be understood within the broader landscape of abdominal wall closure strategies. Compared to monofilament, mesh suture offers distinct biomechanical benefits with early clinical data demonstrating a fascial dehiscence rate of 1.0% that compares favorably to literature-reported rates of 2.6%–3.3% for monofilaments [5, 14, 35]. Mesh suture has also shown early favorable infection-related outcomes. The 90-day SSI rate for mesh suture was 9.0% that is lower than the 13.0% rate reported in a meta-analysis of nearly 500,000 laparotomy patients for open procedures [36]. Perhaps most notable, the chronic mesh-related sinus formation rate was only 0.3%, far lower than the 3.5% rate seen with permanent suture in contaminated wounds [35].

Planar mesh reinforcement remains the current standard for abdominal wall closure during hernia repair, largely due to its demonstrated efficacy in reducing hernia recurrence. Landmark studies, including the PRIMA trial, showed a significant reduction in incisional hernia rates from 21.0% to 12.0% with mesh reinforcement at 2 years [37]. Similarly, Burger et al. demonstrated that mesh has improved outcomes in hernia repair over standard sutures [38]. However, there is great heterogeneity in the literature regarding mesh material, plane of placement, and fixation technique [9–11, 39, 40]. Permanent synthetic mesh has demonstrated the lowest long-term recurrence rates but may be associated with greater risk of mesh-related infections, pain, adhesions, extrusions, fistula formation, and mesh fractures [10, 40]. Although uncommon, central mesh fractures, defined as failure through the body of the mesh rather than at fixation points is being identified with increasing frequency [41]. These central failures can lead to delayed recurrence and may be under-recognized clinically and radiographically.

Permanent synthetic mesh use in contaminated settings is highly debated, even with growing evidence supporting its use in these settings [10, 11, 40]. Biologic mesh has been promoted for use in contaminated settings due to its favorable infection profile. However, studies highlight limitations including recurrence rates exceeding 40.0% and significant costs associated with these materials [42, 43]. As such, biologic mesh offered no clear advantage over synthetic options in this context. Biosynthetic mesh offers an intermediate option, with lower recurrence rates than biologics and comparable early outcomes to permanent synthetics, however early data is limited in its applicability and conclusions [9, 39, 44]. Nevertheless, the quality of the foreign body reaction and scar after full absorption of the biosynthetic material remains unanswered. The original publications with polydioxanone stated with confidence that there was no residual tissue effect upon disappearance of the absorbable filaments, and the same may be true for longer lasting absorbable materials [27]. With this in mind, it may be no surprise that the hernia recurrence rates have been reported up to 20.0% at 5 years using absorbable synthetic mesh [44].

These findings emphasize that no single technique or material universally addresses all clinical scenarios. Rather than replacing planar mesh or conventional suture, mesh suture serves as a complementary adjunct that provides a macroporous implant that reinforces the suture line while preserving native tissue planes. Mesh suture embodies several attributes prioritized in modern abdominal wall reconstruction, including permanent load-sharing support and rapid tissue integration. Ultimately, optimal repair strategy depends not only on patient and wound characteristics but also on surgeon expertise, technique familiarity and institutional resources.

## Discussion and Proposed Clinical Algorithm

**FIGURE 1:**
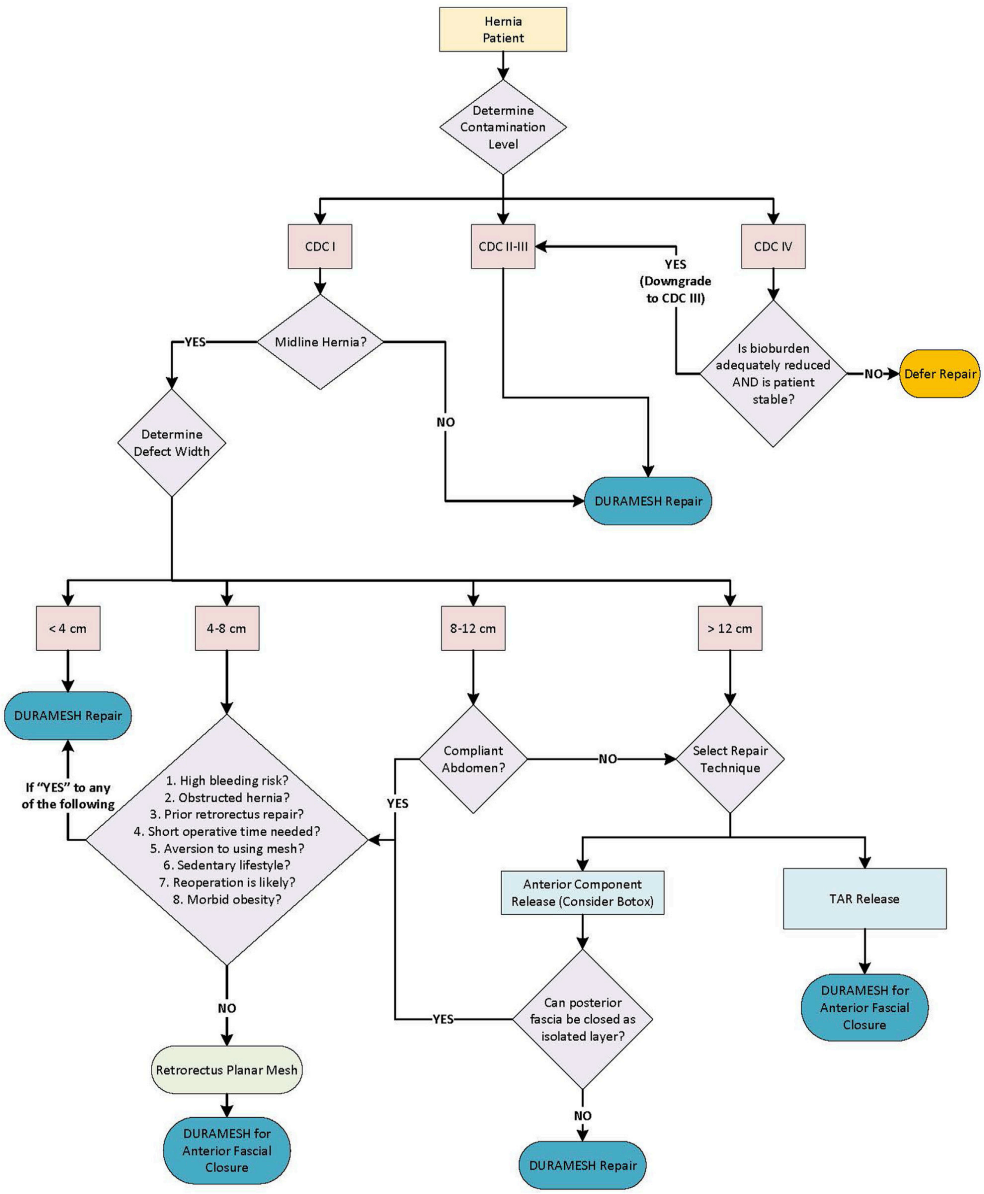
Hernia repair and reconstruction algorithm based on clinical experience.

Based on institutional experiences, mesh suture is used within a structured decision-making framework that considers hernia size and location, patient comorbidities, contamination, and abdominal wall compliance (Figure 1).

### Contamination Status

In clean-contaminated or contaminated (CDC II-III) cases, mesh suture may be favored over planar mesh because it minimizes foreign body burden and preserves vascularity. When fascial defects exceed 8–12 cm or tension is excessive, mesh suture is often combined with perforator-preserving anterior components separation to promote favorable primary closure without additional planar mesh [45]. Preoperative botulinum toxin is another adjunct to increase lateral abdominal wall compliance and reduce suture tension. In dirty/infected wounds (CDC IV), closure is delayed until wound is converted to CDC III (contaminated) with debridement and negative-pressure therapy.

### Defect Location

Non-midline defects, which typically have a higher muscle to fascia ratio, can generally be repaired with mesh suture alone. The highly vascular muscle tissue permits rapid incorporation of the filaments, the mesh suture design resists tearing, and tension relief can be achieved with proper patient positioning on the operating table [46]. This is especially important for flank hernias that often represent a hernia of the internal oblique and transverse abdominis muscles (with an intact external oblique) rather than an abdominal wall denervation that is commonly assumed [47]. The feasibility and durability of mesh suture for non-midline defects without an underlying planar mesh is based on mesh strip outcomes in numerous patients where primary repair is both feasible and facile [46].

### Small Defects

Small defects (<4 cm) such as umbilical hernias, trocar site hernias, and other small defects do not require excessive tension for closure and are optimally closed with mesh suture without additional planar mesh. Umbilical defects are closed transversely, small defects within the rectus muscle (such as ostomy site hernias) are closed vertically along the direction of the muscle fibers, and small hernias lateral to the semilunar line are typically closed obliquely along the dermatome line. In defects >4 cm, patient-specific factors and abdominal wall compliance help determine repair technique.

### Abdominal Wall Compliance and Tension Assessment

Compliant abdomens, such as in patients with low visceral burden (e.g., post-weight loss) or in post-partum females may be closed primarily with mesh suture alone. Not only should the defect be assessed, but the patient should be examined for rectus diastasis. In cases of rectus diastasis with midline hernias, long closures that repair the entire midline are often performed. When tension is moderate and the patient is high demand, a retrorectus mesh is often placed to add additional support to the fascial closure. An alternative for patients who wish to avoid mesh when tension is moderate is to receive preoperative botulinum toxin. High tension midline closures where the defect measures over 12 cm in transverse dimension, for longstanding hernias, and for hernias with a high visceral fat burden require experience and judgement. These patients often cannot have their posterior sheath closed for a standard retrorectus mesh. Therefore, the alternatives include a one-layer mesh suture closure with anterior components release and perforator preservation (with or without preoperative botulinum toxin), or a transversus abdominis (TAR) release that employs a large mesh. Patients that receive a retrorectus mesh can indeed have mesh suture employed to approximate the anterior sheath of the rectus muscles in the midline.

### Patient Specific Risk Considerations

Emergency hernia surgery in non-optimized patients may benefit from a mesh suture closure that will maintain the retrorectus space for another day when intra-abdominal processes and visceral surgery is presumed complete as it has been shown that retrorectus repairs in emergency hernia repairs have a ten-fold odds ratio for complications [48]. Additionally, high-risk patients (e.g., morbidly obese), patients that require postoperative anticoagulation, and/or the frail/older adults may benefit from simpler, faster procedures which avoid dissection required for planar mesh placement that adds operative time, blood loss, extensive exposure, and complexity.

Ultimately, the decision between a planar mesh repair and a mesh suture alone depends on the interplay of tissue tension, compliance, contamination status and patient-specific risk factors. While retrorectus mesh closures have shown low complication rates and hernia recurrence rates at our institution, mesh suture does now play a role in many patients who are not optimal candidates [7, 49].

## Cost Consideration

Formal cost-effectiveness analyses of mesh suture are not yet available. The device cost is higher than traditional suture but comparable to specialty polypropylene meshes and substantially lower lost than biologic or synthetic absorbable mesh materials often used in contaminated settings. Future studies incorporating cost-effectiveness modeling and long-term outcomes will be valuable to better define the economic impact of mesh suture in abdominal wall reconstruction.

## Limitations

Several limitations must be acknowledged. This review is narrative in design, which inherently limits reproducibility and introduces potential selection bias; although a targeted literature search was conducted, study inclusion was not performed using a systematic protocol. The current evidence remains preliminary and consists largely of retrospective, observational, or registry-based studies with follow-up typically less than one-year. Long-term durability, comparative effectiveness, and cost data are not yet available limiting the ability to interpret outcomes as compared to other techniques.

Most published studies originate from a limited number of institutions, including those of the present authors, which may increase the risk of institutional or investigator bias. While full disclosures have been provided, it is important to note that the senior author (G.A.D) has proprietary relationships with the device, but did not participate in data generation, collection, or analysis for the studies summarized here. To strengthen the evidence base, independent, prospective, and multi-institutional trials with long-term follow-up and standardized outcome measures are needed to validate these early findings and clarify mesh suture’s role within the broader spectrum of abdominal wall reconstruction.

## Conclusion

Mesh suture is an emerging reinforcement strategy in abdominal wall reconstruction, developed to address limitations of both suture-only and planar mesh-augmented repairs. Early data suggest mesh suture may offer benefits in cases where traditional techniques fall short. At our institutions, mesh suture has increasingly replaced or complemented both standard suture and planar mesh approaches, however long-term outcomes and comparative studies are needed to validate these findings. As such, mesh suture may occupy a unique niche within a multimodal, individualized approach to fascial closure, warranting continued investigation.
